# Potential for therapeutic use of hydrogen sulfide in oxidative stress-induced neurodegenerative diseases

**DOI:** 10.7150/ijms.36516

**Published:** 2019-09-20

**Authors:** Rubaiya Tabassum, Na Young Jeong

**Affiliations:** 1 Department of Anatomy and Cell Biology, College of Medicine, Dong-A University, 32, Daesingongwon-ro, Seo-gu, Busan, 49201, Korea.; 2 Department of Medicine, Graduate School, Dong-A University, 32, Daesingongwon-ro, Seo-gu, Busan, 49201, Korea.

**Keywords:** central nervous system, hydrogen sulphide, mitochondrial dysfunction, neurodegenerative diseases, oxidative stress

## Abstract

Oxidative phosphorylation is a source of energy production by which many cells satisfy their energy requirements. Endogenous reactive oxygen species (ROS) are by-products of oxidative phosphorylation. ROS are formed due to the inefficiency of oxidative phosphorylation, and lead to oxidative stress that affects mitochondrial metabolism. Chronic oxidative stress contributes to the onset of neurodegenerative diseases, such as Alzheimer's disease (AD), Parkinson's disease (PD), Huntington's disease (HD), and amyotrophic lateral sclerosis (ALS). The immediate consequences of oxidative stress include lipid peroxidation, protein oxidation, and mitochondrial deoxyribonucleic acid (mtDNA) mutation, which induce neuronal cell death. Mitochondrial binding of amyloid-β (Aβ) protein has been identified as a contributing factor in AD. In PD and HD, respectively, α-synuclein (α-syn) and huntingtin (Htt) gene mutations have been reported to exacerbate the effects of oxidative stress. Similarly, abnormalities in mitochondrial dynamics and the respiratory chain occur in ALS due to dysregulation of mitochondrial complexes II and IV. However, oxidative stress-induced dysfunctions in neurodegenerative diseases can be mitigated by the antioxidant function of hydrogen sulfide (H_2_S), which also acts through the potassium (K_ATP_/K^+^) ion channel and calcium (Ca^2+^) ion channels to increase glutathione (GSH) levels. The pharmacological activity of H_2_S is exerted by both inorganic and organic compounds. GSH, glutathione peroxidase (Gpx), and superoxide dismutase (SOD) neutralize H_2_O_2_-induced oxidative damage in mitochondria. The main purpose of this review is to discuss specific causes and effects of mitochondrial oxidative stress in neurodegenerative diseases, and how these are impacted by the antioxidant functions of H_2_S to support the development of advancements in neurodegenerative disease treatment.

## 1. Introduction

Oxygen consumption is essential for cell survival. However, oxygen consumption can cause cell dysfunction and cell death, due to the production of free radicals in mitochondria. Neurodegenerative diseases are caused by excessive free radical generation within neurons, which leads to neuronal cell death in Alzheimer's disease (AD), Parkinson's disease (PD), Huntington's disease (HD), and amyotrophic lateral sclerosis (ALS). Oxidative stress in mitochondria negatively impacts cellular function, as lipids, proteins, and nucleic acids are oxidized by reactive oxygen species (ROS), by-products of the electron transport chain (ETC), and subsequently aggregate in a destructive manner 1. Additionally, there is an absence of protective histone molecules to protect against ROS because they are routinely generated in the inner mitochondrial membrane (IMM) 2. Thus, mitochondrial deoxyribonucleic acid (mtDNA) mutations are caused by excessive ROS formation.

ROS produced in mitochondria comprise hydrogen peroxide (H_2_O_2_), super oxide (O*_•_*_2_^-^) and hydroxyl ion (•OH). In general, oxidative stress occurs when ROS are produced at rates higher than those at which the body can efficiently neutralize reactive metabolites 3. It has been reported that neurodegenerative diseases may occur as a result of mitochondrial dysfunction 3, such as abnormalities in mitochondrial fusion and fission, increased level of cytoplasmic Ca^2+^, DNA mutation, and mitochondrial membrane depolarization. Excessive ROS formation also triggers the accumulation of abnormal proteins that cause neurodegeneration 4. For instance, oxidative changes in mitochondriamay cause protein misfolding in the amyloid-β (Aβ) protein in AD, which results in a wide variety of pathological symptoms 5. Oxidative stress has been linked to PD; mitochondrial fusion is inhibited by the accumulation of α-synuclein (α-syn) protein in PD patients 6. In addition, the mitochondrial proteins, PTEN-induced putative kinase 1(PINK1) and parkin, are both critical for quality control in mitochondria, and are negatively impacted in patients with PD. An expanded level of polyglutamate in huntingtin (Htt) is the major source of oxidative damage in HD 7,8; mtDNA mutations and structural deformities in the mitochondrial genome are responsible for the pathology of ALS. A mutation in superoxide dismutase 1 (SOD1) leads to overproduction of ROS through overexpression of nitric oxide synthase (NOS), as well as abnormal gliosis involving microglial cells;these changes contribute to the pathology of ALS 9.

Notably, the therapeutic effects of hydrogen sulfide (H_2_S) can reduce the detrimental impacts of oxidative stress. The antioxidant functions of H_2_S are exerted by its modifications of enzyme activities, including those of glutathione peroxidase (Gpx), SOD, and catalase (CAT) 10. Gpx acts as intracellular enzyme that converts H_2_O_2_ to lipid peroxide in mitochondria. Gpx is often referred to as selenocysteine peroxide, and has a key regulatory function in the inhibition of lipid peroxidation; therefore, it protects cells from oxidative stress. In humans, eight enzymes, Gpx1-Gpx8, have been identified; among these, Gpx1 is the most abundant, and Gpx enzymes are tetrameric in nature. The antioxidant properties of all Gpx enzymes can be hindered by low expression, and deficiencies of Gpx enzymes have been associated with oxidative stress 11. SOD is a very common antioxidant that catalyzes the dismutation of O*_•_*_2_^-^ to molecular oxygen (O_2_) and increases production of H_2_O_2_. Eventually, H_2_O_2_ decomposes to H_2_O and O_2_ 12. When oxidative stress increases, the SOD concentration also increases. Notably, there are multiple SODs; these include the metalloenzymes, iron (Fe)SOD (homodimer and tetramer forms) and manganese (Mn)SOD (homodimer and homotetramer forms) 13. Simultaneously, CAT reacts efficiently with hydrogen donors, such as phenols or peroxides, to limit the H_2_O_2_ concentration in cells; CAT acts as a first-line antioxidant enzyme by mediating the breakdown of millions of H_2_O_2_ molecules. A high concentration of H_2_O_2_ is reportedly deleterious to cells 14. The principal focus of this review is to describe mitochondrial oxidative stress, oxidative stress-induced mitochondrial dysfunctions that are linked to the onset of age-associated neurodegenerative diseases, and advanced regulatory functions of H_2_S against oxidative stress.

## 2. Oxidative stress and mitochondrial dysfunction

**Figure 1:**
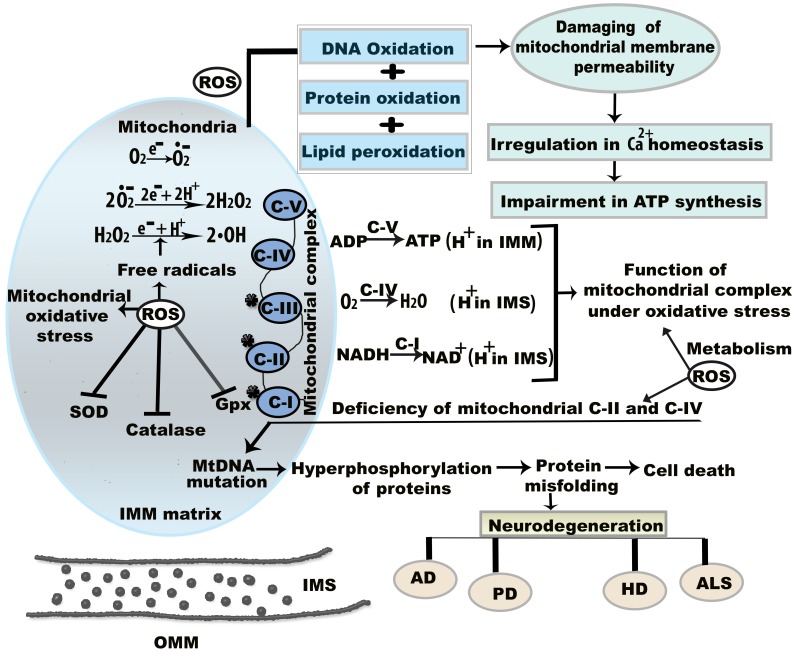
**Molecular mechanism of mitochondrial oxidative stress and dysfunctions.** Oxidative stress and resultant components incite neurodegeneration through three noteworthy causes including (a) mitochondrial dysregulation, excitotoxicity, and protein aggregation (b) mtDNA mutation, and (c) energy depletion. ROS is generated that reduces membrane permeability between OMM and IMM. This permeability difference disturbs ATP synthesis and calcium (Ca^2+^) homeostasis between the membranes. Under oxidative stress condition, superoxide (O*_•_*_2_^-^), hydrogen peroxide (H_2_O_2_), and hydroxyl (•OH) radicals are formed in IMM. Here, the star mark on mitochondrial complex I, II, III indicates that they are more prone to generate free radicals.* Abbreviations*: ETC, electron transport chain; IMM, inner mitochondrial membrane; IMS, intermembrane space; OMM, outer mitochondrial membrane; ROS, reactive oxygen species; NADPH, nicotinamide adenine dinucleotide phosphate; GPx, glutathione peroxidise, SOD, super oxide dismutase; CAT, catalase. ROS is generated that reduces membrane permeability between OMM and IMM.

Mitochondrial dysregulation was first associated with increased ROS formation in a living organism in 1954 15. ROS generation has been related to the onset of age-associated neurodegenerative maladies and cell signaling pathways 16. Although the presence of a moderate level of ROS is advantageous for cellular function, excessive ROS generation leads to oxidative damage to cellular functions and underlying molecular mechanisms (Figure 1) 15. Mitochondria are sources of intracellular ROS, which are formed by mitochondrial complexes I and III of the respiratory chain 17. The metabolic activities of mitochondrial complexes generate oxidative stress by the production of O*_•_*_2_^-^ and H_2_O_2_ (Figure 1). Inhibition or absence of complex I in the respiratory chain causes neuronal apoptosis 18. For example, mitochondrial complex I is inhibited by 1-methyl-4-phenyl-pyridinium, a metabolite of 1-methyl-4-phenyl-1,2,3,6 tetrahydropyridine, which causes cytotoxicity in dopamine neurons 19. Mitochondrial components also show altered function under oxidative stress. Oxidative stress-induced mutations in mtDNA have harmful effects on mitochondrial function over time. mtDNA mutations result in abnormalities in the oxidative phosphorylation process, which manifests as mitochondrial dysfunction through the loss of cellular function and eventual apoptosis 20. In addition, 8-hydroxy-2'-deoxyguanosine is a biomarker of oxidative damage and DNA damage due to free radical attack; this indicates defective mitochondrial respiration and impaired antioxidant enzymes, and suggests that apoptotic cell death is likely to occur 21,22.

During aging, oxidative stress and mitochondrial dysfunction are associated through the erythroid nuclear factor-related factor 2-antioxidant response element (Nrf2-ARE) pathway. Nrf2-ARE is the master regulatory pathway for redox homeostasis 23. In the presence of oxidative stress, Nrf2 binds to the ARE. Nrf2 deficiency impacts antioxidant enzymes, thereby causing impaired regeneration in aged skeletal muscle 24. Coleman *et al.* described that muscle fibers of UCP1-transgenic mice showed impaired mitochondrial respiration. Aged Nrf2 knockout mice reportedly showed increased ROS and 4-hydroxynonenal (4-HNE) in muscle; however, this finding is controversial, as another study reported an altered redox balance due to an increased level of oxidative stress, and stated that there were no clear adverse effects of Nrf2 deficiency 25. Mitochondrial Bcl-2 family proteins and apoptotic Bax proteins also play key roles in extrinsic and intrinsic cell death pathways. Cytochrome *c* releases the Bax protein, which results in apoptosis 26.

## 3. Mitochondrial oxidative stress and neurodegenerative disease

Central nervous system (CNS) functions are related to mitochondrial function. Notably, changes in the mitochondrial genome, abnormalities in mitochondrial dynamics, excessive production of ROS, and accumulation of misfolded protein all might contribute to the onset of neurodegenerative diseases 27. Abnormalities in mitochondrial dynamics and accumulation of metals have been shown to synergistically produce ROS 27. In particular, AD, PD, HD, ALS, and other neurodegenerative diseases are reported to result from ROS-induced mutations in mtDNA 28.

Age-regulated genes may impact biological function by either increasing production of ROS or reducing the availability of ATP, which is fundamental for mitochondrial repair; in addition, the absence of ATP can cause cellular apoptosis 29. Maharjan *et al.* reported that mitochondria act as an important regulator of cellular apoptosis with respect to neurodegeneration. Defects in the mitochondrial ETC system, deficiency in cytochrome oxidase *c*, and differences in mitochondrial membrane potential can cause disruption of energy metabolism and subsequent apoptosis 30. For instance, inhibition of mitochondrial complex I in PD and ALS, complexes II and III in HD, and complexes II and IV in AD stimulate disorganized oxidative phosphorylation and result in apoptosis 31. Furthermore, apoptotic pathways are initiated by caspase activity; caspases are a group of cysteine proteases that regulate apoptosis: caspase-3 was reported to participate in Aβ1-42-induced apoptosis in SH-SY5Y neuronal cells, based on oxidative stress via metallic reaction 32. Normally, oxidative damage to cellular components results in altered catalyst function and protein structure 33.

PD is the most prominent neurodegenerative disorder. At the cellular level, PD is associated with an abundance of ROS that results in modified catecholamine digestion due to either altered mitochondrial ETC function or increased iron deposition in the substantia nigra part compacta (SNpc). Apoptosis then occurs because dopamine neurons experience increased vulnerability 6. Moreover, O*_•_*_2_^-^ radicals are formed as a result of insufficient oxidative phosphorylation in mitochondria which is the principal cause of ROS formation.

In HD, the underlying reason for oxidative damage is the presence of mutant Htt, which contributes to ROS production in both neuronal and non-neuronal cells 34. Iron disorders may underlie oxidative stress in affected cells; these disorders include increased accumulation of ferritin, which is the main form of cellular iron, due to altered iron homeostasis 35. In HD, mutant Htt binds to p53; subsequently, increased levels of p53 and associated transcriptional factors cause increased depolarization of mitochondrial membrane potential 36. SOD1 has generally been identified as a cytoplasmic protein and is located in the outer mitochondrial membrane, intermembrane space, and IMM; SOD1 mutations are suspected to constitute the oxidative stress-induced factor in the onset of ALS. Notably, mutant SOD1 was proposed to result from increased levels of O*_•_*_2_^-^ which can cause oxide to deliver peroxynitrite; this negative feedback system suppresses SOD1 functional capacity 37. SOD1 has generally been identified as a cytoplasmic protein and it is located in the outer membrane of mitochondria (OMM), IMS, and IMM, where SOD1 mutation is considered as the oxidative stress-induced factor in ALS.

H_2_S neutralizes ROS and ROS-induced mitochondrial damage in neurodegenerative diseases, and could be harnessed to achieve progressive therapeutic outcomes for oxidative stress-affected neurons, as described in the following sections.

## 4. H_2_S

### 4.1 Synthetic precursors and metabolism of H_2_S

H_2_S is endogenously produced from pyridoxal phosphate (PLP)-dependent enzymes in mammalian tissues and the normal level of H_2_S for both plasma and tissue is 50-160µM 38. The H_2_S-producing enzymes are cystathionine β synthase (CBS), cystathionine γ lyase (CSE), cysteine aminotransferase, and a zinc-dependent enzyme, 3-mercaptopyruvate sulfurtransferase (3MST) 39. Among these enzymes, CBS is highly expressed in the hippocampus and cerebellum, which are components of the CNS. CBS is a precursor protein, which is regulated by transforming growth factor α and cyclic adenosine monophosphate 40.CSE is generally considered to be present in endothelial cells, but has recently been observed in microglial cells, cerebellar granular neurons, and spinal cord 38. CSE produces H_2_S, as well as pyruvate and ammonia byproducts, by catalyzing L-cysteine. Chiku *et al*. reported that CSE-mediated α and β-elimination of L-cysteine produced a yield of 70% of the physiological level of H_2_S 41. However, approximately 90% of the physiological level of H_2_S is derived from α, γ-elimination of homocysteine. In the presence of PLP, CSE activity is reduced because of increased Ca^2+^ concentration 42. An additional source of H_2_S is bound sulfane sulfur, where intracellular sulfur is stored in the absence of GSH and cysteine 43. Bound sulfur is produced by 3MST; L-cysteine and α-ketoglutarate combine to serve as the source of 3MST 43.

H_2_S metabolism occurs during mitochondrial oxidation. Sulfide is oxidized to elemental sulfur in the presence of quinine oxidoreductases (SQRs). During reduction of cysteine disulfides, SQRs produce cysteine disulfides and persulfide groups 44. Each persulfide is oxidized by sulfur deoxygenase (SDO), thus producing sulfite (H_2_SO_3_) 44. Oxygen consumption is necessary during H_2_S metabolism (Table 1) and one mole of oxygen is consumed for each mole of H_2_S oxidized in the ETC system 45.

In contrast to CBS and CSE, 3MST is primarily present in kidney, liver, and cardiac cells, where it is mainly located in mitochondria; H_2_S is also produced in mitochondria. Recent studies have shown that, in the presence of 3MST, brain homogenates of CBS knockout mice produced levels of H_2_S similar to those of wild-type mice.

### 4.2 Antioxidant and antiapoptotic functions of H_2_S

H_2_S provides enzymatic antioxidant function by mediating the activities of Gpx, SOD, and CAT. Gpx is the most common H_2_S-mediated antioxidant derivative, which acts through reduction of peroxides 46. The antioxidant function of Gpx involves production of non-biological thiols when •OH radicals are present; these are less likely to cause oxidative damage than H_2_O_2_, which is highly reactive and has deleterious effects 47,48.

**Figure 2:**
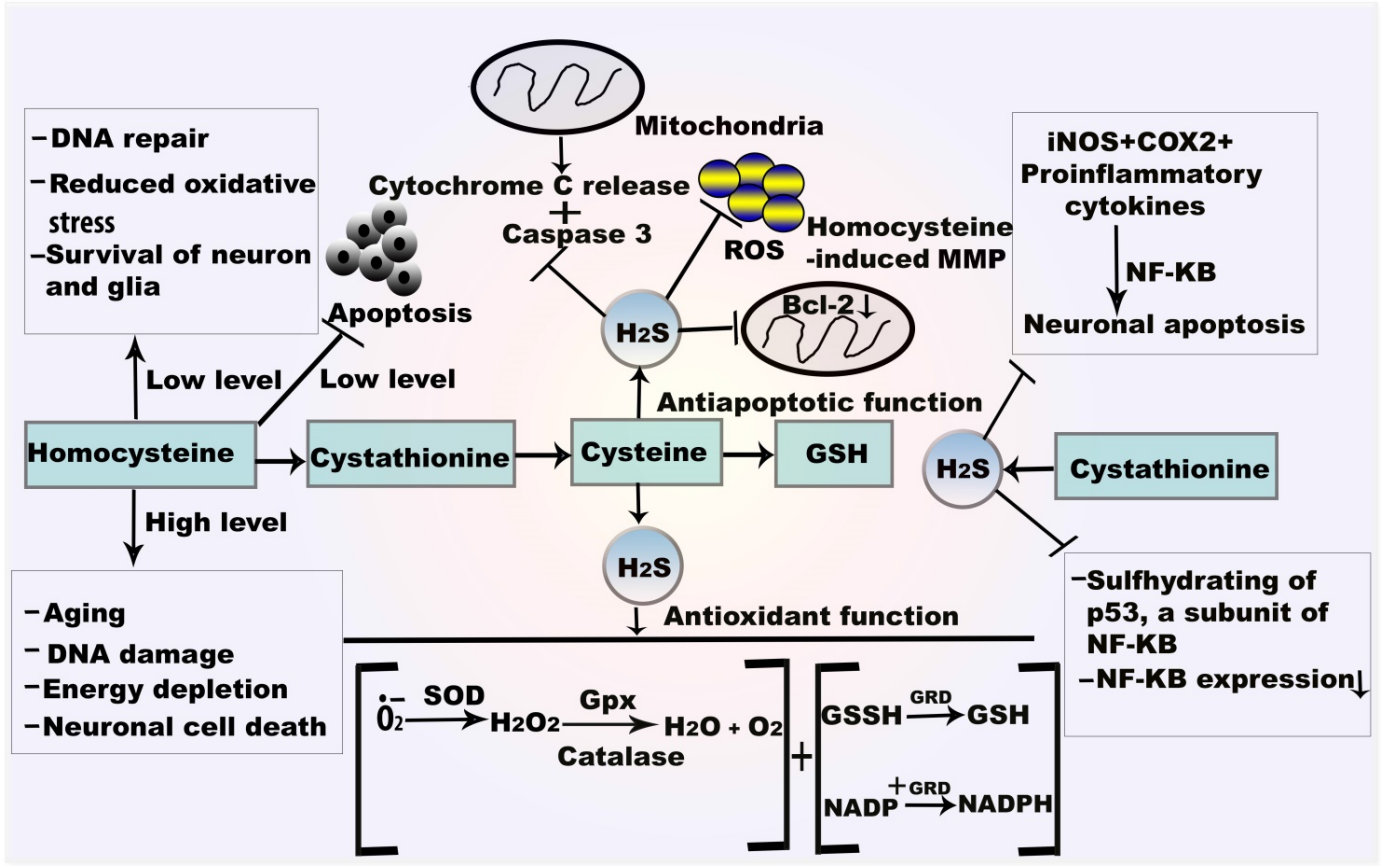
**Mechanism of H_2_S in autoxidation and antiapoptosis**.GSH reacts with oxygen free radical which directly form the thiol radical and later GSSH. SOD catalyses the dismutation of O*_•_*_2_^-^ and converted to H_2_O and O_2_. H_2_O_2_ is also attenuated by the catalysis of CAT and Gpx. H_2_S also provides antiapoptotic function by NF-κBand caspase 3. H_2_S from CSE plays role in sulfhydrating the p65 subunit of NF-κBat cysteine 38. *Abbreviations*: GSSH, oxidized glutathione; GRD, glutathione reductase; NF-κB, nuclear factor kappa B.

SODs play major antioxidant roles, especially against O*_•_*_2_^-^. Generally, SODs exhibit three isoforms: cytoplasmic copper (Cu)/zinc (Zn) SOD (SOD1), mitochondrial MnSOD (SOD2), and extracellular Cu/Zn SOD (SOD3). O*_•_*_2_^-^ is modified by SOD enzymes during cell signaling 49. The importance of each SOD as an antioxidative agent is illustrated by the pathophysiology of CNS degenerative diseases. Initially, SOD converts O*_•_*_2_^-^ to H_2_O_2_; then, H_2_O_2_ is converted to H_2_O by CAT or Gpx (Figure 2). Increased SOD1 activity elevates H_2_O_2_ levels, such that they become toxic 49. The catalytic activities of SOD1 involve reduction and reoxidation of Cu and Mn at the active site of the enzyme; these comprise regulators of O*_•_*_2_^-^ proportion 50. SOD1 and SOD2 both reduce the incidence of H_2_O_2_-induced oxidative damage 51.

CAT catalyzes H_2_O_2_ to O_2_ and H_2_O. H_2_O_2_ participates in H_2_S metabolism in hypoxia, suggesting that H_2_O_2_ is an effective electron receptor in this reaction 52. Generally, CAT generates H_2_S from carbonyl sulfide, cysteine, GSH, or oxidized GSH, and serves as a sulfur oxidase or sulfur reductase. In the presence of the CAT inhibitor, sodium azide (NaN_3_), H_2_O_2_ significantly expedites H_2_S metabolism (Figure 2). Apoptotic signals by caspase-1 and caspase-3 are sequentially activated in SOD1 mutant mice: caspase-1 is active at an early stage and caspase-3 is active in the final stage of cell death.

## 5. H_2_S functions in Ca^2+^ and K_ATP_ion channels

**Figure 3:**
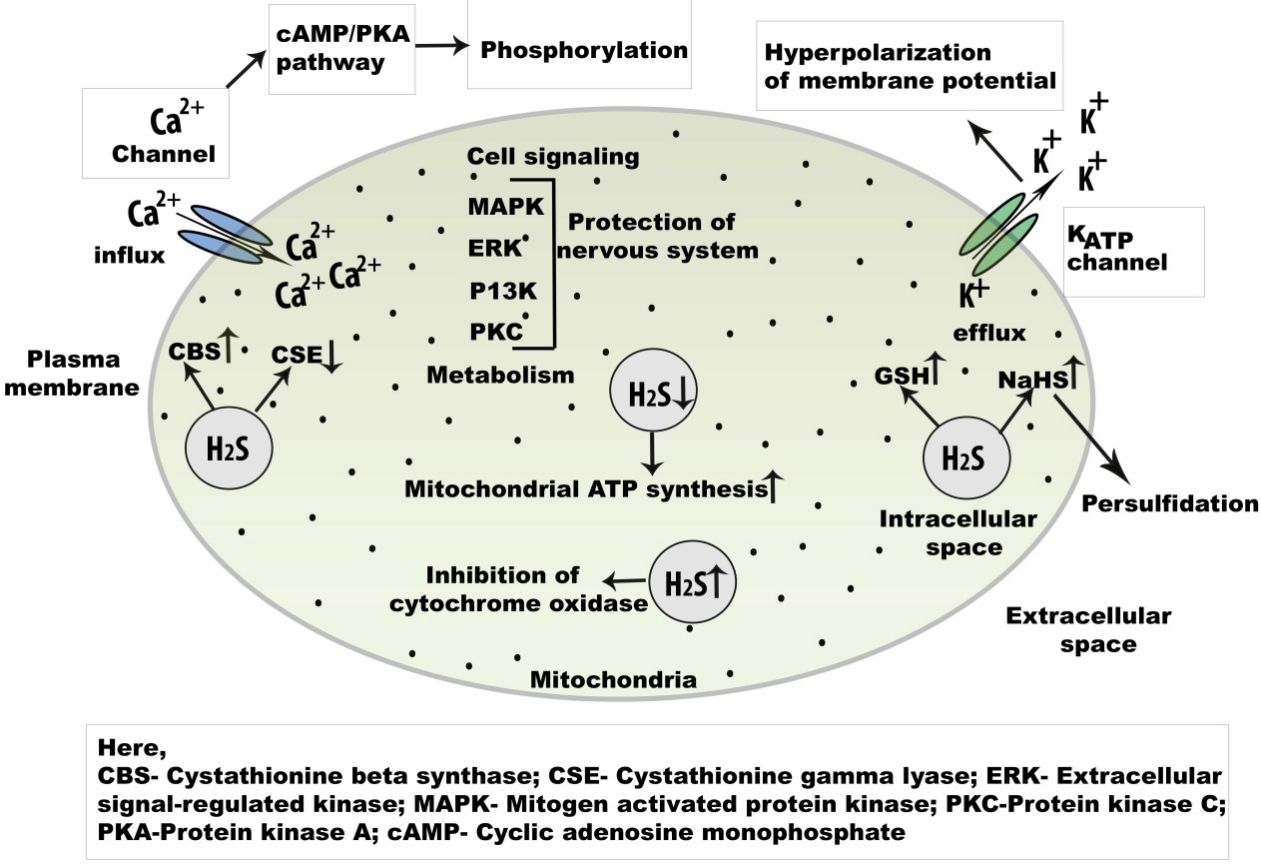
**Cell signaling regulation of endogenous H_2_S in the central nervous system**. Physiologically, H_2_S is an important signaling molecule and regulate L and T type Ca^2+^ channel. As a key regulator of Ca^2+^signaling in neuron, the concentration of NaHSis increased.The activation of cAMP/PKA may open the Ca^2+^ channel initiates phosphorylation which helps to open several Ca^2+^ channels. Besides, champ/PKA pathway, cell signaling is mediated by MAPK, ERK, P13K, and PKC pathways. *Abbreviations*: NaHS, sodium hydrogen sulphide; cAMP, cyclic adenosine monophosphate; MAPK, mitogen activated protein kinase; PKA, protein kinase A; PKC, protein kinase C; ERK, extracellular regulatory kinase.

In the CNS, intracellular Ca^2+^ plays key roles in both normal and pathological signaling. H_2_S has been found to promote increased Ca^2+^levels in neurons, astrocytes, and microglial cells. In serotonergic neurons, a biphasic response is produced by H_2_S during depolarization 39. In addition, plasma membrane voltage-gated channels are activated by H_2_S, including T-type channels, whereas L-type Ca^2+^ channels are expressed in neurons and secrete both neurohormones and neurotransmitters 53. The action of H_2_S on L-type Ca^2+^ channels were demonstrated through a study of the effects of the L-type channel-specific blocker, nifedipine, in rat cerebellar granule neurons 38. Recently, H_2_S was discovered to enhance stimulation of Ca^2+^ entry via L-type channels; this Ca^2+^ was shown to participate in neurotransmitter release and gene expression. Furthermore, T-type channels have a role in somatic pain; they act against high-voltage gated channels or have a low activation threshold. T-type Ca^2+^ channels are present in hippocampal CA1 cells, thalamic neurons, and Purkinje cells in the cerebellum 54. Additionally, H_2_S activates the Ca_v_3.2T-type channel isoform, which regulates rhythmic neuronal function and neuronal differentiation 55. Furthermore, physiological concentrations of H_2_S mobilize intracellular Ca^2+^ storage in various cells (Figure 3). Intracellular Ca^2+^ storage participates in long-term potentiation in neurons and facilitates the release of glutamate from presynaptic terminals 55.

K_ATP_ channels are considered primary molecular targets for H_2_S. Generally, K_ATP_ channels aid in neurotransmitter release from presynaptic neurons, control seizures, and provide neuroprotection in hypoxic conditions 56. H_2_S hyperpolarizes neurons in the CA1 by K^+^ efflux through ATP-dependent K_ATP_ channels, which are opened as a result of oxidative glutamate toxicity 57. By opening K_ATP_ channels, H_2_S increases GSH levels. H_2_S is also present in immortalized mouse hippocampal cells, where it facilitates the opening of ATP-dependent K_ATP_ channels 58. Overall, Ca^2+^ channels and K_ATP_ channels contribute to H_2_S-mediated cell signaling.

## 6. Neuroprotective potential of hydrogen sulfide as antioxidant

**Figure 4:**
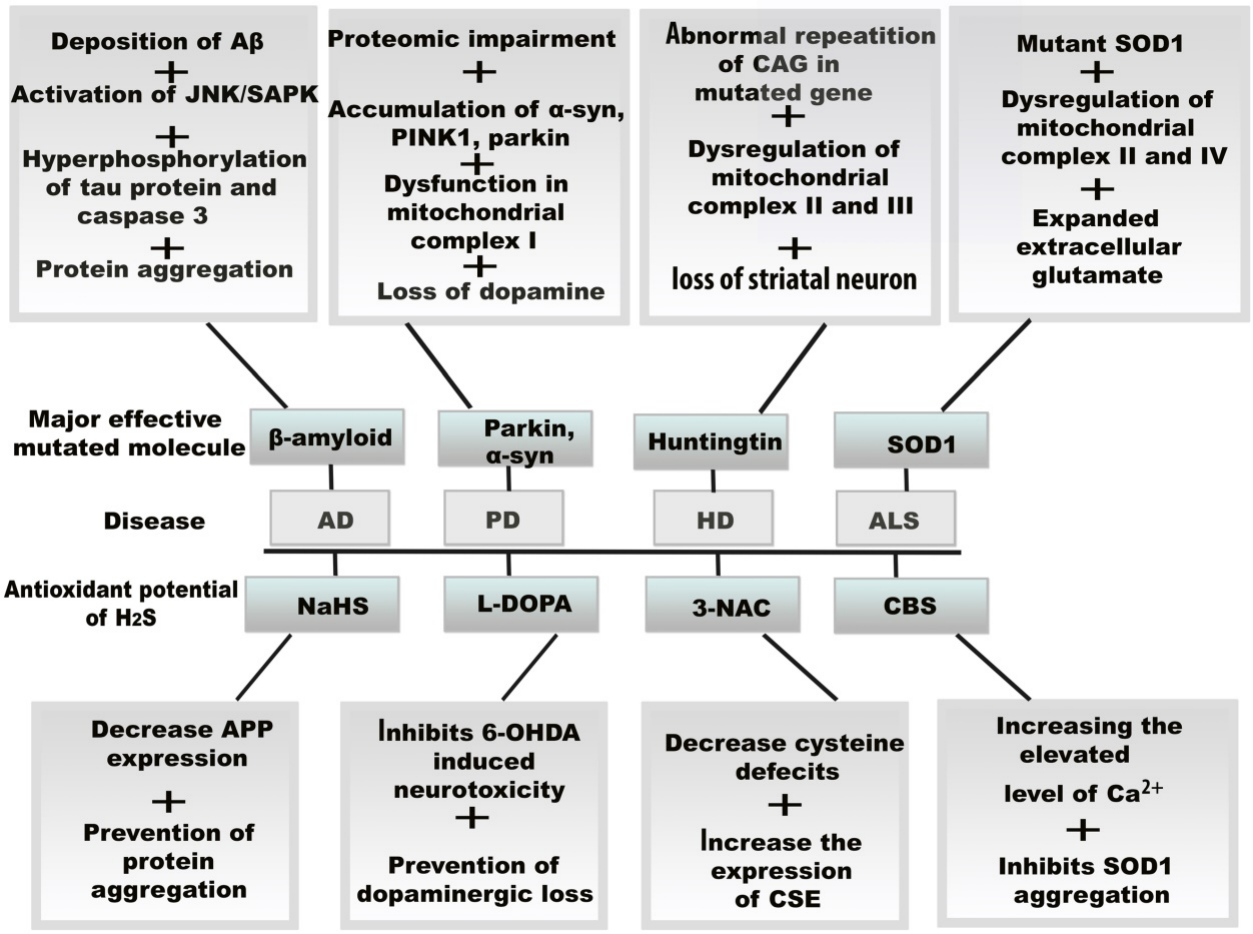
** Resultant effects of mitochondrial oxidative stress and therapeutic potential of H_2_S in neurodegenerative diseases.** The vital role of H_2_S against oxidative stress, the amplifying H_2_S level induces several molecular changes in neurodegenerative diseases by the increasing and decreasing the enzymes including CBS, CSE, and 3-3MST. H_2_S also exerts its antioxidant function by binding drug molecule and activating protein precursors.*Abbreviations*: Aβ, amyloid β; AD, Alzheimer's disease; ALS, amyotrophic lateral sclerosis; CBS, cystathionine β synthase; CSE, cystathionine γ lyase; 3MST, 3-mercaptopyruvate sulfurtransferase; HD, Huntington disease; PD, Parkinson's disease; L-DOPA, levodopa; 6-OHDA, 6-hydroxydopamine.

Progressive loss of neurons is responsible for neurodegenerative disease.H_2_S acts as an effective antioxidant to fight against oxidative stress in neurodegenerative diseases, through the action of H_2_S donors or enzymatic antioxidant mechanisms (Figure 4).

### 6.1 AD

As a gasotransmitter, the antioxidant function of H_2_S in AD is vital. General hallmarks of AD include the mutation of amyloid precursor protein (APP) and aggregation of both Aβ and tau proteins. According to a clinical study, elevated homocysteine levels were decreased and excitatory amino acid transporter 3 (EAAT3/EAAC1) inhibited the GSH level 59. Increased expression of H_2_S through Nrf2 indicates that MDA and 4-HNE are generated as a result of reduced homocysteine. Here, Nrf2 is the central mediator of redox balance. In addition, intraperitoneal injection of sodium hydrosulfide (NaHS) in experimental APP/PS1 mice causes downregulation of beta-secretase 1 (BACE1) through the p13/Akt pathway; notably, BACE1 is responsible for the production of Aβ peptides. NaHS is an H_2_S donor that has been shown to decrease Aβ plaques and increase spatial memory 60. Moreover, NaHS reduces phosphorylation of APP and tau proteins at critical sites and diminishes morphological damage, including damage mediated by neuronal death 61. NaHS acts against homocysteine-induced cognitive dysfunction 62. Parker *et al.* showed that mitochondrial complexes II and IV were deficient in hippocampal neurons of AD patients. WhenH_2_O_2_causes mitochondrial membrane damage and excess Ca^2+^, cellular GSH regulates intramitochondrial protein thiols and selective membrane permeability 63.

CBS in the CNS and CSE in the cardiovascular system are sources of endogenous H_2_Sgeneration. In the brain, 3MST is also a significant source of H_2_S. Reduced expression levels of CBS and 3MST have been observed in neurons, such as rat PC12 cells, upon exposure to NaN_3_; conversely, H_2_S suppresses NaN_3_-induced oxidative stress 64. Moreover, dysfunction of CBS in the trans-sulfuration pathway may reduce H_2_S generation in AD. Furthermore, S-adenosyl-L-methionine, an activator of CBS, is lower in AD brains than in those of normal individuals.

### 6.2 PD

H_2_S has also a potential role in the neuromodulation of PD. To eliminate oxidative elements, continuous Gpx action is needed to recycle reduced GSH to its oxidized form. Overexpression of CBS or H_2_S donors provides neuroprotection against 6-hydroxydopamine-induced neurotoxicity 62. H_2_S signaling is affected by the E3-ubiquitin ligase, parkin,which is a misfolded protein in PD. The main targets for sulfhydration on parkin are cys95, cys59, and cys182 62. Importantly, 6-hydroxydopamineis widely regarded as the factor responsible for the death of dopaminergic neurons through dopamine uptake transporters. Two H_2_S donors, ACS84 and ACS50, have the greatest contributions as antioxidants. ACS84 exerts L-3,4-dihydroxyphenylalanine (L-DOPA)-mediated effects in PD, such that it can penetrate the blood brain barrier (BBB) and release H_2_S 65. Because homocysteine is a precursor of H_2_S, the plasma level of homocysteine can be used to assess the effects of H_2_S in PD in the context of a particular drug treatment. L-DOPA is a potent anti-PD medication that alleviates symptoms by maintaining the dopamine concentration at the synapse and reducing motor fluctuations 66. Approximately 15-20% of patients do not respond to L-DOPA therapy and may show adverse profiles after long-term therapy 67. According to a clinical study by Obeid et al., 87 patients showed high levels of total homocysteine (t-homocysteine) with increased levels of APP and α-synuclein 68. A case-control study from Nigeria described 80 individuals, 40 of whom were healthy controls, while the remaining 40 were PD patients of the same age group with high levels of homocysteine who received L-DOPA mediated treatment 69. L-DOPA mediated changes in homocysteine have revealed key regulatory functions in oxidative stress-induced neurological damage 70.

### 6.3 HD

Polyglutamate repeats in the Htt protein cause transcriptional dysfunction in motor neurons in the HD mouse model and human HD brain during cysteine metabolism when CSE is depleted in cell culture. Reduced CSE expression causes lower levels of cysteine; as a result, H_2_S levels are reduced and ROS generation is increased in mitochondria (Figure 4) 62,71.

CBS might be a useful target for the treatment of neurodegeneration in HD. In a recent study, hyperhomocystinuria was observed in HD patients, as compared to controls, because the mutated Htt protein modulates homocystinuria-induced CBS activity. Moreover, HD patients are affected by both cardiovascular and cerebrovascular diseases 72. Andrich et al. reported the concentration (17.7 µmol/l) of homocysteine in 34 HD patients treated with antidepressants, neuroleptics, benzodiazepines, and/or tetrabenazine, compared to the concentrations in untreated HD patients (12.6 µmol/l) and 73 healthy controls (13.3 µmol/l). In that study, untreated HD patients were less severely affected and had shorter disease duration than the treated patients, which indicates a positive correlation between the plasma level of homocysteine and untreated HD 73. In HD, cytosolic CSE is depleted at the transcriptional level and could reflect the translocation of CSE to insoluble aggregates. In Q111 cells, CSE was depleted to a similar extent in both supernatant and particulate fractions. Generally, striatal Q111 cells showed greater susceptibility to H_2_O_2_ stress. mHtt also reportedly binds to and inhibits specific protein 1 (SP1); CSE depletion in HD seems to reflect inhibition of Sp1 by mHtt, leading to reduced CSE transcription 74.

### 6.4 ALS

H_2_S can counteract oxidative modification through insoluble SOD1 aggregation, which is a common feature of ALS. Free cysteine in SOD, specifically at Cys111, is responsible for SOD1 mutation in ALS (Figure 4). However, H_2_S provides an antioxidant function through elevation of CBS 62. The G93A (fALS) mouse model reportedly exhibited increased H_2_S generation in tissues and spinal cord, along with increased intracellular Ca^2+^ levels. In addition, elevated H_2_S was also identified in the CSF fluid of ALS patients, which suggests gasotransmitter signaling in ALS 62. Posttranslational modification of SOD1 may enable formation of toxic aggregates. In a phase III clinical trial of ALS patients, ceftriaxone upregulated the GLT-1 (EAAT-2) glutamate transporter, this may have corrected glutamate levels. Another phaseIII clinical trial reported that high doses of methylcobalamin (vitamin B-12) reduced homocysteine levels in ALS patients 75.

An investigation of the levels of CBS-containing lanthionine (a thioether analogue of cysteine) in ALS showed that LanCL1 levels were elevated by three-fold in SOD1^G93A^ mice. In contrast, immunoblot analysis of spinal cord lysates from mice overexpressing wild-type human SOD1 indicated altered LanCL1 expression 76. Therefore, CBS-targeting treatment in ALS is not yet clearly defined as a therapeutic approach. Further investigation is necessary regarding CBS-targeting treatment in ALS.

In summary, H_2_S exhibits protective effects in neurodegenerative diseases through antioxidant functioning. Although H_2_S neutralizes harmful oxidative modification in neurodegenerative diseases, additional *in vivo* studies are needed to elucidate molecular mechanisms in oxidative stress.

## 7. Pharmacological effects of H_2_S

The pharmacological effects of H_2_S are exerted by inhibition of H_2_S/H_2_S donors or augmentation of endogenous H_2_S; many experimental models have demonstrated the protective effects of H_2_S or potential targets of H_2_S donors in neuromodulation, hypertension, and inflammation 44. Although some experimental studies show harmful effects of H_2_S, these are controversial. For instance, sulfide salts comprise donors of H_2_S that may have H_2_S-independent effects. In contrast, lower H_2_S levels may lead to reduced expression levels of CBS and CSE inhibitors, known as genetic inhibition. CBS and CSE inhibitors may also cause H_2_S-independent effects through genetic inhibition, such as cysteine deficiency due to hyperhomocysteinemia and enhanced GSH synthesis. Finally, abnormalities have been observed in mice in which CBS, CSE, or 3MST have been knocked out 77.

Sulforaphane (SF) is a derivative of H_2_S, synthesized from isothiocyanate, which causes enhanced expression of CBS and CSE 78,79. Moreover, *in vivo* experiments have shown that cell signaling pathways, such as p38 MAPK and JNK, are activated by SF. After absorption, SF is conjugated with GSH by glutathione s-transferase 79. In terms of bioavailability, the plasma concentration and metabolic components increased and reached the highest levels after 1 and 3 hours, respectively. The urinary excretion of SF drugs within 12-14 hours reflects rapid elimination 80. Experimental studieshave shown that SF-Cys and SF-N-acetyl cysteine (NAC) also exert some bioactivity. In neurodegenerative disorders, SFis observed as combined metabolites (e.g., SF-GSH, SF-Cys, and SF-NAC). SF has also shown poor ability to cross the BBB, but reaches the CNS very rapidly 79.

Among cysteine derivatives, S-propyl-cysteine (SPC), S-allyl-cysteine (SAC), and S-proparglycysteine (SPRC) are good substrates from which CBS and CSE can produce H_2_S. SPC, SAC, and SPRC are administered to reduce lipid peroxidation and increase the activation of GSH, SOD, and Gpx 81. SPRC reduces NF-κB activity, decreases ROS production, and inhibits the TNF-α-induced inflammatory response 82. According to Wang *et al.,* SPC, SAC, and SPRC all increased H_2_S generation by at least two-fold at the carbon terminal, as measured in homogenized rat ventricles. H_2_S increased in the hippocampus of lipopolysaccharide-treated rats in a dose-dependent manner 44. A major pathway by which H_2_S protects against cellular damage is the Nrf2-dependent signaling pathway 83.

The pharmacological activity of H_2_S-releasing drugs in cell signaling has been assessed by *in vitro* studies. Studies of H_2_S-releasing drug *in vivo* are more difficult than *in vitro* studies due to physiological and pathological conditions. To determine more fully the pharmacological effects of H_2_S-releasing drugs, further research is necessary.

## 8. Conclusion

Neurons have the capacity for cell-cell communication. When this communication fails, symptoms of neurodegenerative diseases occur. As discussed above, mitochondrial damage is connected to the pathogenesis of neurodegenerative diseases. Protein damage, DNA mutations, and membrane permeability are vulnerable to oxidative damage, which plays a pathogenic role in AD, PD, HD, and ALS. Generally, mitochondrial homeostasis is maintained by various protein structures and functions are not identical among proteins. However, it remains unclear how the harmful effects of oxidative stress are mediated in specific neuronal diseases. Identification of specific disease-related proteins, to discern relationships between specific proteins and mitochondrial oxidative stress, can be achieved through further broad studies.

Mitochondrial dysfunction due to ROS formation is a prominent feature of neurodegenerative diseases, dysfunctional characteristics should be mitigated through the protective effects of the H_2_S gasotransmitter. Furthermore, the details of cellular responses of H_2_S to ROS-mediated oxidative stress must be explored. To identify the therapeutic potentials of H_2_S, particular enzyme inhibitors are needed, based on their abilities to augment gasotransmitter synthesis. The cytoprotective effect of H_2_S as a signaling molecule against ROS, as well as cell-specific enzymatic activities (e.g., CBS, CSE, and 3MST), may add further protection against neurodegenerative diseases.

## Figures and Tables

**Table 1:** Synthetic precursors and metabolites of hydrogen sulfide (H_2_S)

H_2_S produced enzymes	Substrates	Synthesized products
**H_2_S synthesis**		
CSE	L-Cysteine	Pyruvate, H_2_S, ammonia
CSE	L-Cystathionine	L-Cysteine, α-ketobutyrate, ammonia
CSE	L-Homocysteine	α -ketobutyrate, H_2_S, ammonia
CBS	L-Homocysteine, L-cysteine	L-Cystathionine, H_2_O
CBS, CSE	L-Homocysteine, L-cysteine	L-Cystathionine, H_2_S
CBS, CSE	L-Cysteine,	L-lanthionine, H_2_S
CBS,CSE	L-Homocysteine	L-Homolanthionine, H_2_S
CAT	L-Cysteine, glutamate	3-Mercaptopyruvate, α-ketogluterate
3MST	3-mercaptopyruvate	3-Mercaptopyruvate
DAO	D-cysteine	Pyruvate, H_2_S
**H_2_S Metabolism**		
SQR	H_2_S	SQR persulfide
Rhodanese	Oxidized GSH, SO_3_^2-^	GSH, SSO_3_^2-^
Thiosulfate reductase	SSO_3_^2-^, GSH	SO_3_^2-^, H_2_S
Sulfite oxidase	SO_3_^2-^	SO_4_^2-^

CSE, cystathionine γ lyase; CBS, cystathionine β synthase; CAT, cysteine aminotransferase; 3MST, 3-mercaptopyruvate sulfurtransferase; DAO, diamine oxidase; SQR, sulfide quinone oxidoreductase; GSH, glutathione.

## References

[1] Hassan E, Kahilo K, Kamal T, El-Neweshy M, Hassan M (2019). Protective effect of diallyl sulfide against lead-mediated oxidative damage, apoptosis and down-regulation of CYP19 gene expression in rat testes. Life Sci.

[2] Chiang S-C, Meagher M, Kassouf N, Hafezparast M, McKinnon PJ, Haywood R (2017). Mitochondrial protein-linked DNA breaks perturb mitochondrial gene transcription and trigger free radical-induced DNA damage. Sci Adv.

[3] Georgieva E, Ivanova D, Zhelev Z Mitochondrial Dysfunction and Redox Imbalance as a Diagnostic Marker of “Free Radical Diseases.” Anticancer Res. 2017;37(10):5373-81.

[4] Zhelev Z, Bakalova R, Aoki I, Lazarova D, Saga T (2013). Imaging of superoxide generation in the dopaminergic area of the brain in Parkinson's disease, using mito-TEMPO. ACS Chem Neurosci.

[5] Gella A, Durany N (2009). Oxidative stress in Alzheimer disease. Cell Adhes Migr.

[6] Blesa J, Trigo-Damas I, Quiroga-Varela A, Jackson-Lewis VR (2015). Oxidative stress and Parkinson's disease. Front Neuroanat.

[7] Manoharan S, Guillemin GJ, Abiramasundari RS, Essa MM, Akbar M, Akbar MD (2016). The Role of Reactive Oxygen Species in the Pathogenesis of Alzheimer's Disease, Parkinson's Disease, and Huntington's Disease: A Mini Review. Oxid Med Cell Longev.

[8] Wu B, Jiang M, Peng Q, Li G, Hou Z, Milne GL (2017). 2,4 DNP improves motor function, preserves medium spiny neuronal identity, and reduces oxidative stress in a mouse model of Huntington's disease. Exp Neurol.

[9] Barber SC, Shaw PJ (2010). Oxidative stress in ALS: Key role in motor neuron injury and therapeutic target. Free Radic Biol Med.

[10] E Birben, U.Sahiner, C.Sackesen (2012). Oxidative stress and antioxidant defense. World allergy Organization Journal.

[11] Forgione MA, Weiss N, Heydrick S, Cap A, Klings ES, Bierl C (2002). Cellular glutathione peroxidase deficiency and endothelial dysfunction. Am J Physiol Circ Physiol.

[12] Reczek CR, Chandel NS (2015). ROS-dependent signal transduction. Curr Opin Cell Biol.

[13] Muid KA, Karakaya HÇ, Koc A (2014). Absence of superoxide dismutase activity causes nuclear DNA fragmentation during the aging process. Biochem Biophys Res Commun.

[14] Ighodaro OM, Akinloye OA (2017). First line defence antioxidants-superoxide dismutase (SOD), catalase (CAT) and glutathione peroxidase (GPX): Their fundamental role in the entire antioxidant defence grid. Alexandria J Med.

[15] Ahmad W, Ijaz B, Shabbiri K, Ahmed F, Rehman S (2017). Oxidative toxicity in diabetes and Alzheimer's disease: mechanisms behind ROS/ RNS generation. J Biomed Sci.

[16] Höhn A, Weber D, Jung T, Ott C, Hugo M, Kochlik B (2017). Happily (n)ever after: Aging in the context of oxidative stress, proteostasis loss and cellular senescence. Redox Biol.

[17] Boengler K, Kosiol M, Mayr M, Schulz R, Rohrbach S (2017). Mitochondria and ageing: role in heart, skeletal muscle and adipose tissue. J Cachexia Sarcopenia Muscle.

[18] Kujoth GC, Hiona A, Pugh TD, Someya S, Panzer K, Wohlgemuth SE (2005). Mitochondrial DNA mutations, oxidative stress, and apoptosis in mammalian aging. Science.

[19] Essawy SS, Tawfik MK, Korayem HE (2017). Effects of adenosine receptor antagonists in MPTP mouse model of Parkinson's disease: mitochondrial DNA integrity. Arch Med Sci.

[20] Mikhed Y, Daiber A, Steven S (2015). Mitochondrial oxidative stress, mitochondrial DNA damage and their role in age-related vascular dysfunction. Int J Mol Sci.

[21] Vieira G de LT, Lossie AC, Lay DC, Radcliffe JS, Garner JP (2017). Preventing, treating, and predicting barbering: A fundamental role for biomarkers of oxidative stress in a mouse model of Trichotillomania. PLoS ONE.

[22] Dalmasso G, Zapata PAM, Brady NR, Hamacher-Brady A (2017). Agent-based modeling of mitochondria links sub-cellular dynamics to cellular homeostasis and heterogeneity. PLoS ONE.

[23] Mary Ann Liebert, Madison Avenue Larchmon (2005). Molecular Mechanism of Nrf2 Activation byOxidative Stress. Antioxidnt and Redox signaling.

[24] Kitaoka Y, Takeda K, Tamura Y, Fujimaki S, Takemasa T, Hatta H (2016). Nrf2 deficiency does not affect denervation-induced alterations in mitochondrial fission and fusion proteins in skeletal muscle. Physiol Rep.

[25] Narasimhan M, Hong J, Atieno N, Muthusamy VR, Davidson CJ, Abu-Rmaileh N (2014). Nrf2 deficiency promotes apoptosis and impairs PAX7/MyoD expression in aging skeletal muscle cells. Free Radic Biol Med.

[26] Chang C-C, Huang T-Y, Chen H-Y, Huang T-C, Lin L-C, Chang Y-J (2018). Protective Effect of Melatonin against Oxidative Stress-Induced Apoptosis and Enhanced Autophagy in Human Retinal Pigment Epithelium Cells.

[27] Giulia N, Simona S, Maria D, Simona R (2017). Oxidative Stress, Mitochondrial Abnormalities and Proteins Deposition: Multitarget Approaches in Alzheimer's Disease. Curr Top Med Chem.

[28] Lee H-C, Wei Y-H (2007). Oxidative stress, mitochondrial DNA mutation, and apoptosis in aging. Exp Biol Med.

[29] Federico A, Cardaioli E, Da Pozzo P, Formichi P, Gallus GN, Radi E (2012). Mitochondria, oxidative stress and neurodegeneration. J Neurol Sci.

[30] Cardoso SM, Rego AC, Penacho N, Oliveira CR (2004). Apoptotic cell death induced by hydrogen peroxide in NT2 parental and mitochondrial DNA depleted cells. Neurochem Int.

[31] Tiiman A, Palumaa P, Tõugu V (2013). The missing link in the amyloid cascade of Alzheimer's disease-metal ions. Neurochem Int.

[32] Gao J, Wang L, Liu J, Xie F, Su B, Wang X (2017). Abnormalities of Mitochondrial Dynamics in Neurodegenerative Diseases. Antioxidants.

[33] Shah SZA, Zhao D, Hussain T, Yang L (2017). The role of unfolded protein response and mitogen-activated protein kinase signaling in neurodegenerative diseases with special focus on prion diseases. Front Aging Neurosci.

[34] Sayre LM, Perry G, Smith MA (2008). Oxidative stress and neurotoxicity. Chem Res Toxicol.

[35] Luthi-Carter R, Cha J-HJ (2003). Mechanisms of transcriptional dysregulation in Huntington's disease. Clin Neurosci Res.

[36] Barnham KJ, Masters CL, Bush AI (2004). Neurodegenerative diseases and oxidative stress. Nat Rev Drug Discov.

[37] Kasarskis EJ, Lindquist JH, Coffman CJ, Grambow SC, Feussner JR, Allen KD (2009). Clinical aspects of ALS in Gulf War veterans. Amyotroph Lateral Scler.

[38] Munaron L, Avanzato D, Moccia F, Mancardi D (2013). Hydrogen sulfide as a regulator of calcium channels. Cell Calcium.

[39] Tan BH, Wong PT-H, Bian J-S (2010). Hydrogen sulfide: a novel signaling molecule in the central nervous system. Neurochem Int.

[40] Enokido Y, Suzuki E, Iwasawa K, Namekata K, Okazawa H, Kimura H (2005). Cystathionine β-synthase, a key enzyme for homocysteine metabolism, is preferentially expressed in the radial glia/astrocyte lineage of developing mouse CNS. FASEB J.

[41] Chiku T, Padovani D, Zhu W, Singh S, Vitvitsky V, Banerjee R (2009). H2S biogenesis by human cystathionine γ-lyase leads to the novel sulfur metabolites lanthionine and homolanthionine and is responsive to the grade of hyperhomocysteinemia. J Biol Chem.

[42] Mikami Y, Shibuya N, Ogasawara Y, Kimura H (2013). Hydrogen sulfide is produced by cystathionine γ-lyase at the steady-state low intracellular Ca2+ concentrations. Biochem Biophys Res Commun.

[43] Shibuya N, Tanaka M, Yoshida M, Ogasawara Y, Togawa T, Ishii K (2009). 3-Mercaptopyruvate sulfurtransferase produces hydrogen sulfide and bound sulfane sulfur in the brain. Antioxid Redox Signal.

[44] Kashfi K, Olson KR (2013). Biology and therapeutic potential of hydrogen sulfide and hydrogen sulfide-releasing chimeras. Biochem Pharmacol.

[45] Hildebrandt TM, Grieshaber MK (2008). Three enzymatic activities catalyze the oxidation of sulfide to thiosulfate in mammalian and invertebrate mitochondria. FEBS J.

[46] Battin EE, Brumaghim JL (2009). Antioxidant activity of sulfur and selenium: a review of reactive oxygen species scavenging, glutathione peroxidase, and metal-binding antioxidant mechanisms. Cell Biochem Biophys.

[47] Perron NR, Hodges JN, Jenkins M, Brumaghim JL (2008). Predicting how polyphenol antioxidants prevent DNA damage by binding to iron. Inorg Chem.

[48] Xie Z-Z, Liu Y, Bian J-S (2016). Hydrogen sulfide and cellular redox homeostasis. Oxid Med Cell Longev.

[49] Fukai T, Ushio-Fukai M (2011). Superoxide dismutases: role in redox signaling, vascular function, and diseases. Antioxid Redox Signal.

[50] Rae TD, Schmidt PJ, Pufahl RA, Culotta VC, O'halloran T V (1999). Undetectable intracellular free copper: the requirement of a copper chaperone for superoxide dismutase. Science (80- ).

[51] Beyer Jr WF, Fridovich I (1987). Effect of hydrogen peroxide on the iron-containing superoxide dismutase of Escherichia coli. Biochemistry.

[52] Olson KR (2013). Hydrogen sulfide as an oxygen sensor. Clin Chem Lab Med.

[53] Nagai Y, Tsugane M, Oka J-I, Kimura H (2004). Hydrogen sulfide induces calcium waves in astrocytes. FASEB J.

[54] Huguenard JR (1998). Low-voltage-activated (T-type) calcium-channel genes identified. Trends Neurosci.

[55] Oheim M, Kirchhoff F, Stühmer W (2006). Calcium microdomains in regulated exocytosis. Cell Calcium.

[56] Walewska A, Szewczyk A, Koprowski P (2018). Gas Signaling Molecules and Mitochondrial Potassium Channels. Int J Mol Sci.

[57] Qu K, Lee SW, Bian JS, Low CM, Wong PTH (2008). Hydrogen sulfide: Neurochemistry and neurobiology. Neurochem Int.

[58] Li L, Rose P, Moore PK (2011). Hydrogen Sulfide and Cell Signaling. Annu Rev Pharmacol Toxicol.

[59] Hodgson N, Trivedi M, Muratore C, Li S, Deth R (2013). Soluble oligomers of amyloid-β cause changes in redox state, DNA methylation, and gene transcription by inhibiting EAAT3 mediated cysteine uptake. J Alzheimer's Dis.

[60] Giuliani D, Ottani A, Zaffe D, Galantucci M, Strinati F, Lodi R (2013). Hydrogen sulfide slows down progression of experimental Alzheimer's disease by targeting multiple pathophysiological mechanisms. Neurobiol Learn Mem.

[61] Vandini E, Ottani A, Zaffe D, Calevro A, Canalini F, Cavallini GM (2019). Mechanisms of hydrogen sulfide against the progression of severe Alzheimer's disease in transgenic mice at different ages. Pharmacology.

[62] Paul BD, Snyder SH (2018). Gasotransmitter hydrogen sulfide signaling in neuronal health and disease. Biochem Pharmacol.

[63] Bains JS, Shaw CA (1997). Neurodegenerative disorders in humans: the role of glutathione in oxidative stress-mediated neuronal death. Brain Res Rev.

[64] Gao C, Chang P, Yang L, Wang Y, Zhu S, Shan H (2017). Neuroprotective effects of hydrogen sulfide on sodium azide-induced oxidative stress in PC12 cells.

[65] Predmore BL, Lefer DJ, Gojon G (2012). Hydrogen Sulfide in Biochemistry and Medicine. Antioxid Redox Signal.

[66] Group PS (2004). Levodopa and the progression of Parkinson's disease. N Engl J Med.

[67] Guin D, Mishra MK, Talwar P, Rawat C, Kushwaha SS, Kukreti S (2017). A systematic review and integrative approach to decode the common molecular link between levodopa response and Parkinson's disease. BMC Med Genomics.

[68] Obeid R, Schadt A, Dillmann U, Kostopoulos P, Fassbender K, Herrmann W (2009). Methylation Status and Neurodegenerative Markers in Parkinson Disease. Clin Chem.

[69] Lu H, Liu X, Deng Y, Qing H (2013). DNA methylation, a hand behind neurodegenerative diseases. Front Aging Neurosci.

[70] Shin JY, Ahn Y-H, Paik M-J, Park HJ, Sohn YH, Lee PH (2012). Elevated homocysteine by levodopa is detrimental to neurogenesis in parkinsonian model. PLoS One.

[71] Li X, Valencia A, McClory H, Sapp E, Kegel KB, DiFiglia M (2012). Deficient Rab11 activity underlies glucose hypometabolism in primary neurons of Huntington's disease mice. Biochem Biophys Res Commun.

[72] Zoccolella S, Martino D, Defazio G, Lamberti P, Livrea P (2006). Hyperhomocysteinemia in movement disorders: current evidence and hypotheses. Curr Vasc Pharmacol.

[73] Andrich J, Saft C, Arz A, Schneider B, Agelink MW, Kraus PH (2004). Hyperhomocysteinaemia in treated patients with Huntington's disease homocysteine in HD. Mov Disord Off J Mov Disord Soc.

[74] Paul BD, Sbodio JI, Xu R, Vandiver MS, Cha JY, Snowman AM (2014). Cystathionine γ-lyase deficiency mediates neurodegeneration in Huntington's disease. Nature.

[75] Pratt AJ, Getzoff ED, Perry JJP (2012). Amyotrophic lateral sclerosis: update and new developments. Degener Neurol Neuromuscul Dis.

[76] Chung CHY, Kurien BT, Mehta P, Mhatre M, Mou S, Pye QN (2007). Identification of lanthionine synthase C-like protein-1 as a prominent glutathione binding protein expressed in the mammalian central nervous system. Biochemistry.

[77] Cao X, Ding lei, Xie Z-Z, Yang Y, Whiteman M, Moore PK (2018). A review of hydrogen sulfide synthesis, metabolism and measurement: Is modulation of hydrogen sulfide a novel therapeutic for cancer?. Antioxidants Redox Signal.

[78] Morroni F, Sita G, Djemil A, D'Amico M, Pruccoli L, Cantelli-Forti G (2018). Comparison of adaptive neuroprotective mechanisms of sulforaphane and its interconversion product erucin in *in vitro* and *in vivo* models of Parkinson's disease. J Agric Food Chem.

[79] Tarozzi A, Angeloni C, Malaguti M, Morroni F, Hrelia S, Hrelia P (2013). Sulforaphane as a potential protective phytochemical against neurodegenerative diseases.

[80] Veeranki OL, Bhattacharya A, Marshall JR, Zhang Y (2013). Organ-specific exposure and response to sulforaphane, a key chemopreventive ingredient in broccoli: implications for cancer prevention. Br J Nutr.

[81] Wang Q, Wang X-L, Liu H-R, Rose P, Zhu Y-Z (2010). Protective effects of cysteine analogues on acute myocardial ischemia: novel modulators of endogenous H2S production. Antioxid Redox Signal.

[82] Pan LL, Liu XH, Zheng HM, Yang HB, Gong QH, Zhu YZ (2012). S-propargyl-cysteine, a novel hydrogen sulfide-modulated agent, attenuated tumor necrosis factor-α-induced inflammatory signaling and dysfunction in endothelial cells. Int J Cardiol.

[83] Sestito S, Nesi G, Pi R, Macchia M, Rapposelli S (2017). Hydrogen Sulfide: A Worthwhile Tool in the Design of New Multitarget Drugs. Front Chem.

